# Electroacupuncture on the Scalp over the Motor Cortex Ameliorates Behavioral Deficits Following Neonatal Hypoxia-Ischemia in Rats via the Activation of Neural Stem Cells

**DOI:** 10.3390/life10100240

**Published:** 2020-10-14

**Authors:** Da Hee Jung, Malk Eun Pak, Hong Ju Lee, Sung Min Ahn, Young Ju Yun, Yong-Il Shin, Hwa Kyoung Shin, Seo-Yeon Lee, Byung Tae Choi

**Affiliations:** 1 Department of Korean Medical Science, School of Korean Medicine, Pusan National University, Yangsan 50612, Gyeongnam, Korea; jjj262999@pusan.ac.kr (D.H.J.); cleargirl46@naver.com (M.E.P.); hongju120@pusan.ac.kr (H.J.L.); julie@pusan.ac.kr (H.K.S.); 2 Graduate Training Program of Korean Medicine for Healthy-Aging, School of Korean Medicine, Pusan National University, Yangsan, Gyeongnam 50612, Korea; 3 Korean Medical Science Research Center for Healthy-Aging, Pusan National University, Yangsan 50612, Gyeongnam, Korea; isdly@pusan.ac.kr; 4 Department of Integrative Medicine, School of Korean Medicine, Pusan National University, Yangsan 50612, Gyeongnam, Korea; mdkmdyun@pusan.ac.kr; 5 Department of Rehabilitation Medicine, Research Institute for Convergence of Biomedical Science and Technology, Pusan National University, Yangsan 50612, Gyeongnam, Korea; rmshin01@gmail.com; 6 Department of Pharmacology, Wonkwang University School of Medicine, Iksan 54538, Jeonbuk, Korea

**Keywords:** alternating current stimulation, hypoxia-ischemia, corpus callosum, subventricular zone, dentate gyrus

## Abstract

Electroacupuncture (EA) therapy via alternating current stimulation on the scalp over the motor cortex is used for the treatment of brain disorders. Perinatal hypoxia-ischemia (HI), a brain injury in newborns, leads to long-term neurologic complications. Here, we investigated whether EA could promote functional improvements and neurogenesis in a neonatal HI rat model. A neonatal HI rat model was induced by permanent ligation of the left carotid artery in postnatal day 7 pups. EA for neonatal HI rats was performed at 2 Hz (1, 3, or 5 mA; 20 min) from 4–6 weeks after birth. HI rats undergoing EA had improved motor and memory function, with the greatest improvement after 3 mA EA. The corpus callosum was significantly thicker and showed a significant increase in proliferating astrocytes in the 3 mA EA group. We observed proliferating cells and a greater number of newly developed neurons and astrocytes in the subventricular zone and dentate gyrus of the 3 mA EA group than in those of the HI group. These results suggest that EA promotes functional improvements following neonatal HI assault via the proliferation and differentiation of neural stem cells. This effect was the strongest after 3 mA EA, suggesting that this is the optimal treatment dose.

## 1. Introduction

Perinatal hypoxia-ischemia (HI) is the most prominent cause of brain injury, disability, and death in infants, accounting for 23% of infant mortality worldwide and affecting 0.7–1.2 million infants annually [1,2]. Perinatal HI restricts oxygen and blood supply, which damages brain cells. Perinatal HI leads to long-term neurologic complications such as mild behavioral deficits, severe seizures, mental retardation, and/or cerebral palsy in newborns [3]. HI-associated brain injury in preterm newborns is linked to a high susceptibility for neural stem cell loss, which is dependent on the maturational stage of the brain [4,5].

HI, during the perinatal period, is one cause of cerebral palsy. Intrauterine asphyxia is the most common cause of hypoxic injury, which is characterized by insufficient blood supply caused by clotting of placental arteries, placental abruption, or inflammatory processes, amongst others [6]. Non-progressive demyelination, occurring in cerebral palsy, resulting in severe neurology-related disabilities, such as sensorimotor deficits and cognitive impairments throughout development and into adulthood [7]. There are no restorative or well-established therapies for cerebral palsy [8]; however, many therapeutic strategies have been investigated to improve its functional recovery. 

It is hypothesized that understanding the balance between injury and endogenous repair in neuronal disorders can aid in elucidating potential therapeutic strategies for brain disorders, such as replacement therapy with neural stem/progenitor cells, increasing the potential to rescue the brain from damage and restore motor function [9,10,11]. Endogenous neural stem cells are potential therapeutic targets; however, the number of newly generated cells from neural stem cells is very small and localized to specific brain regions; therefore, appropriate interventions may be necessary for this to become a suitable therapeutic strategy [11,12].

Engrafted alternating current stimulation by fine filiform needle, known as electroacupuncture (EA), is widely used clinically to treat brain disorders in Oriental medicine [13]. In addition, it is accepted as a common complementary therapy for the treatment of stroke [13,14]. EA can ameliorate motor dysfunction and cognitive impairment in ischemic brain disorders by enhancing the proliferation and differentiation of neural stem cells [15,16,17]. We previously reported that acupoint-based EA enhanced the proliferation and differentiation of neural stem cells in mouse focal cerebral ischemia [15,16,17] and in the prolonged cerebral hypoperfusion mouse model [15,16,17]. We also reported that in a cerebral palsy-like model, EA (2 Hz, 1 mA) at two acupoints, Baihui (GV 20) and Zusanli (ST 36), is more effective in achieving functional recovery when combined with other rehabilitation treatments, such as treadmill training or constraint-induced movement therapy [14,18]. Scalp acupuncture, developed from traditional acupuncture and a modern anatomical understanding of cerebral cortex organization, penetrates specific areas of the scalp, which correspond to different cortical regions, using a fine needle [19,20]. In this way, EA is similar to transcranial current stimulation: safe, simple, and inexpensive to administer. In addition, the needle can be used to reach more sites [21]; therefore, scalp EA stimulates a wider area on the scalp over the motor cortex.

In this study, we hypothesized that scalp EA treatment over the motor cortex could effectively recover behavioral deficits following HI, especially sensorimotor function, in a cerebral palsy-like rat model. We applied scalp EA using three intensities (1, 3, and 5 mA) in a widely-used animal model of cerebral palsy, the hypoxic-ischemic Vannucci model [22]. We performed behavioral testing for sensorimotor and memory deficits and immunofluorescence staining to measure the proliferation and differentiation of neuronal stem cells in the corpus callosum, subventricular zone, and dentate gyrus to evaluate the effect of scalp EA.

## 2. Materials and Methods 

### 2.1. Animals

Pregnant Sprague–Dawley rats at embryonic day 17 (E17) were obtained from DooYeol Biotech (Seoul, Korea) and housed at 22 °C, under an alternating 12 h light–dark cycle. The Institutional Animal Care and Use Committee of Pusan National University approved all the experiments in the current study (PNU-2016-1099), and all experiments were performed in accordance with the relevant guidelines and regulations.

### 2.2. Neonatal Hypoxia–Ischemia Model

Neonatal HI was induced in male pups (11–17 g) on postnatal day 7 under 2.0% isoflurane anesthesia (Choongwae, Seoul, Korea) using a model VIP 3000 calibrated vaporizer (Midmark, Orchard Park, OH, USA). Briefly, the left carotid artery was exposed and permanently ligated using 5-0 surgical silk. After surgery, the pups recovered for 1 h with their mother and then were placed in a humidified hypoxic chamber (ProOx oxygen animal chamber, BioSpherix, Redfield, NY, USA) for 1.5 h. The incubator was maintained with 8% oxygen at 36 °C. The sham group did not undergo the surgery and were not exposed to hypoxic conditions. However, they were separated from their mother during both the surgery and hypoxic incubation, and then returned to their mother until weaned completely. HI rats were randomly divided into four treatment groups and underwent EA stimulation.

### 2.3. Electroacupuncture Treatment

**Figure 1:**
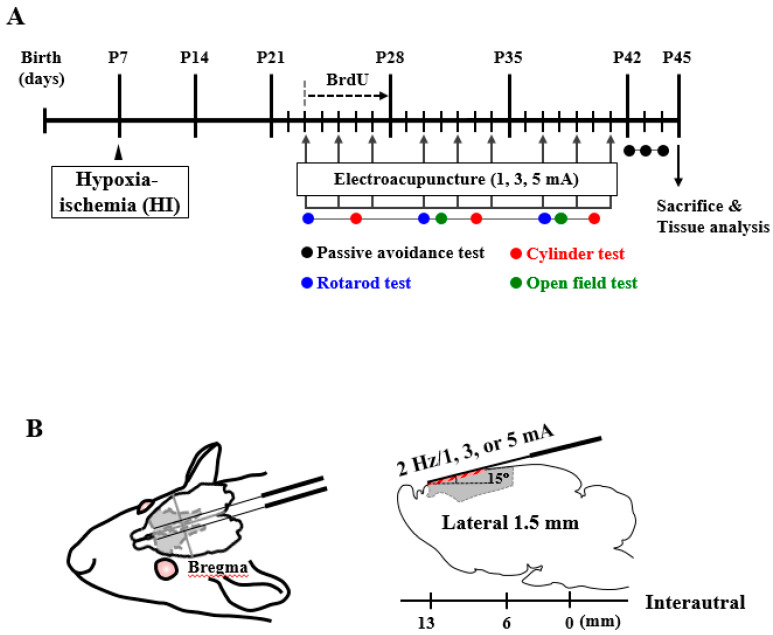
Outline of the experimental protocol and stimulating points. (**A**) Timeline for electroacupuncture (EA) on the scalp over the motor cortex and behavioral tests. Neonatal hypoxia-ischemia (HI) was induced in rat male pups (11–17 g) on postnatal day 7. Rats were stimulated electrically at 2 Hz (1, 3, or 5 mA; 20 min), 3 times a week for 3 weeks. (**B**) Two stainless needles were inserted bilaterally below the subcutaneous tissue of the scalp (approximately 15°: lateral, 1.5 mm; interaural, 6–13 mm) at the primary (M1) and secondary (M2) motor cortex (gray area).

For EA stimulation, HI rats were anesthetized lightly (1.5% isoflurane in air) to minimize stress during EA stimulation. Both sham and HI only group, which did not undergo EA stimulation, were also anesthetized (1.5% isoflurane in air) to exclude the influence of anesthesia. Two stainless steel needles (diameter, 0.2 mm) were inserted bilaterally below the subcutaneous tissue of the scalp (approximately 15°: lateral, 1.5 mm; interaural, 6–13 mm) at the primary (M1) and secondary (M2) motor cortex (gray area). Rats were stimulated electrically at 2 Hz (1, 3, or 5 mA; 20 min), 3 times a week for 3 weeks (Figure 1) using a Pulsemaster Multichannel Stimulator SYS-A300 electrical stimulator (World Precision Instruments, Berlin, Germany).

### 2.4. Brdu Labeling

Rats underwent intraperitoneal (ip) injection with 5-bromodeoxyuridine (BrdU; B5002-5G; 50 mg·kg^−1^; Sigma-Aldrich, Merck KGaA, Darmstadt, Germany), once a day, for 6 days (P23–P28) to label proliferating cells in the brain (Figure 1).

### 2.5. Open Field Test

Rats were assessed in an open field apparatus to evaluate both spontaneous locomotor behavior and anxiety. The open field (60 × 60 × 30 cm) was divided into the center (30 × 30 cm) and surrounding zones. Rats were placed in an individual box in the center, following a 5-min habituation in a black square box. During the 20 min test, total distance (cm), velocity (cm·s^−1^), and entries in the center zone (number) were measured using a SMART v2.5.18 video tracking system (Panlab, S.L.U., Barcelona, Spain), under low light intensity (<50 lx) in a quiet room.

### 2.6. Cylinder Test

The cylinder test was performed to measure forelimb movement and to assess functional recovery from the unilateral brain lesion. Briefly, rats were placed in a transparent Plexiglas cylinder (diameter, 20 cm; height, 30 cm), and the number of limb-wall contacts was counted for the ipsilateral (to the lesion site) and contralateral forelimbs, and support with both forelimbs against the wall was also evaluated during lateral exploration. Data were presented as scores of limb-use asymmetry using the following Equation (1):

(1) $$\frac{\mathrm{right}\;\mathrm{contacts}+(\mathrm{both}\;\mathrm{contacts}\;\text{×}\;0.5)}{\mathrm{total}\;\mathrm{number}\;\mathrm{of}\;\mathrm{contacts}}\;\times \;100$$

### 2.7. Rotarod Test

The accelerated rotarod test was used to assess motor coordination and balance after HI (Panlab S.L.U., Barcelona, Spain). Rats received a training session on the rotarod set at a constant speed of 4 rpm with 10 min rest before testing. Following this, a single baseline trial on the accelerating rotarod was conducted. Briefly, the spindle increased in speed from 4–40 rpm, over 5 min. Rats underwent 3 trials per day. The time spent on the rod before falling off was recorded for each trial, and mean time spent on the rod was calculated from all the trials.

### 2.8. Passive Avoidance Test

The passive avoidance test provides an indication of short-term learning and memory. This test (Med Associates Inc., St. Albans, VT, USA) consisted of three sessions. Illuminated (20 × 20 × 25 cm) and dark (20 × 20 × 25 cm) compartments were separated by a sliding door. Each rat was placed on the safe platform for training trials. The time taken for the rat to enter the dark compartment was measured. After training, when the rat entered the dark compartment, the door closed automatically and a single inescapable scrambled foot shock (0.8 mA, 3 s) was delivered through the grid floor. During the final session, the procedure was repeated until the latency to enter the dark box was ≥300 s.

### 2.9. Immunofluorescence Staining

Following the behavioral tests, rats were anesthetized with 8% chloral hydrate (50 mg·kg^−1^; C8383-100G, Sigma-Aldrich, St. Louis, MO, USA) and received transcardial perfusion with saline, following by 4% paraformaldehyde (PFA) in phosphate buffered saline (PBS). Brains were removed and fixed in 4% PFA at 4 °C for 4 h, and then immersed in 30% sucrose for 72 h at 4 °C for scryoprotection. The isolated brains were frozen using a frozen medium (BBC Biochemical, Mount Vernon, WA, USA) and sliced (30 µm thickness) in accordance with the coronal coordinates in the rat brain atlas: interaural 9.20–10.20 mm, bregma 0.2–1.2 mm (corpus callosum and subventricular zone) and interaural 4.70–5.70 mm, bregma −4.30–−3.30 mm (dentate gyrus) using a CM3050 cryostat (Leica Microsystems, Wetzlar, Germany). After incubation in blocking buffer (1 × PBS/1% bovine serum albumin/0.3% triton X-100) for 1 h at room temperature, sections were incubated with the following primary antibodies overnight in PBS at 4 °C: BrdU (Bromodeoxyuridine, 1:500, MCA2483, Bio-Rad, Hercules, CA, USA); CNPase (2′,3′-Cyclic-nucleotide 3′-phosphodiesterase, 1:100, D83E10, Cell Signaling, Danvers, MA, USA); GFAP (Glial fibrillary acidic protein, 1:500, MAB360, Millipore Corporation, Billerica, MA, USA); NeuN (neuronal nuclei, 1:500, ABN73, Millipore Corporation); MBP (Myelin Basic Protein, 1:500, ab40390, Abcam, Cambridge, UK). Sections were washed with PBST (phosphate buffered saline with 0.1% tween) and incubated with fluorescent secondary antibodies (1:100, A11001 or A11037; Invitrogen, Carlsbad, CA, USA) for 2 h at room temperature (approximate 25 °C). The sections were mounted on slides using a mounting medium (Vector Laboratories, Burlingame, CA, USA) and imaged using a fluorescence microscope (Carl Zeiss Imager M1, Carl Zeiss AG, Oberkochen, Germany). For each rat, a total amount of six coronal-sectioned slides were analyzed by measuring the thickness of middle of CC (one spot) using the IMT i-Solution Inc. 10.1 (17TH-5989 Walter Gage Rd., Vancouver, BC, Canada) image analysis software. As such, one average of thickness was generated for each group of rats. The number of positive cells was counted at one field in a certain volume of 1.57 mm^2^ in the CC and 0.33 mm^2^ in the SVZ and DG. Morphological analysis and quantification of positive cells were conducted in a blinded manner using the IMT i-Solution Inc. 10.1 (17TH-5989 Walter Gage Rd., Vancouver, BC, Canada) image analysis software.

### 2.10. Nissl Staining

Tissue slices (30 µm thickness) were stained with 0.1% fast cresyl violet acetate (C5042, Sigma-Aldrich, St. Louis, MO, USA). Slices were cleared under xylene for 20 min after dry in the air, then rehydrated for 2 min using 100% ethanol to 50% ethanol. After staining in cresyl violet for 2 min and a progressive dehydration protocol procedure. Slices were cleared in xylene for 5 min before covering with mounting medium (ZC0123, Vector Laboratories, Burlingame, CA, USA). Images were taken with ZEISS Stemi 305 Stereo Microscope (Carl Zeiss AG, Oberkochen, Germany).

### 2.11. Data Analyses

All data are expressed as mean ± standard error of the mean (SEM). The normality of the data was first tested using the Shapiro-Wilk test. All data was normally distributed. Data were analyzed in a blinded manner to the phenotype using the SigmaPlot statistical program Version 11.0 (Systat Software, San Jose, CA, USA). Statistical analysis of data was performed using a one-way analysis of variance for repeated measures, followed by the Student-Newman-Keuls test comparison. Statistical significance was set at *P* < 0.05.

## 3. Results

### 3.1. Effects of EA Treatment on Motor and Memory Functions Following Hypoxia-Ischemia

**Figure 2:**
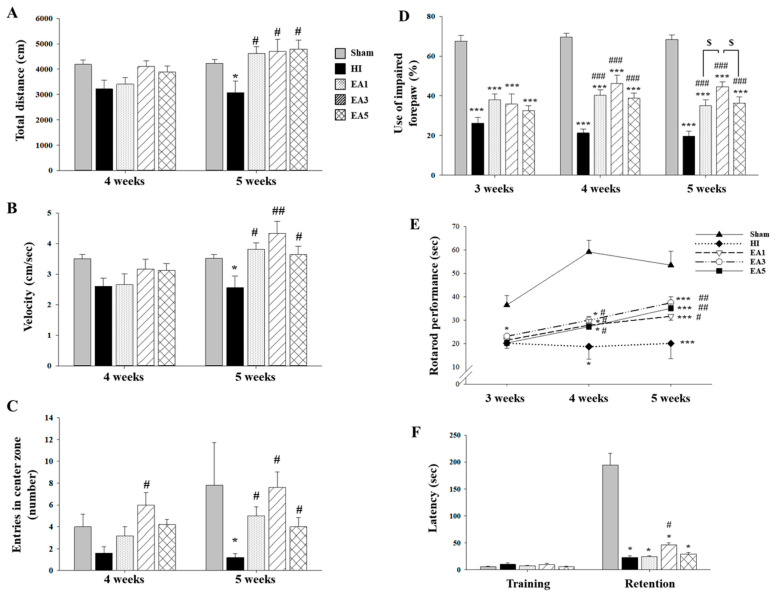
Effect of EA on motor function with anxiety-like responses and memory function after HI. (**A**) The parameters of total distance traveled (n = 6), (**B**) mean velocity (n = 6), and (**C**) entries in the center zone (number, n = 5) were evaluated by an open field test (day 24 and 31 after HI). (**D**) Cylinder test (day 19, 26 and 33 after HI), (**E**) Rotarod test (day 16, 23, and 30 after HI), and (**F**) Passive avoidance test (day 35–37 after HI). HI alone (HI), HI + 1 mA (EA1), HI + 3 mA (EA3), and HI + 5 mA (EA5). Spontaneous locomotor function was higher in all EA treatment groups than in the HI group. In addition, EA groups showed a decrease in the time spent in the center zone. This effect was the strongest in the EA3 group. EA3 ameliorated memory impairment when compared with the results in the HI group. The behavior tests measured in 3 weeks, 4 weeks and 5 weeks after HI. Data are presented as mean ± SEM (n = 5 or 6 per group). * *p* < 0.05 and *** *p* < 0.001 vs. sham, ^#^ *p* < 0.05, ^##^ *p* < 0.01 and ^###^ *p* < 0.001 vs. HI. ^$^ *p* < 0.05 vs. EA3.

To investigate the effect of EA on the scalp over the motor cortex in a rat hypoxia-ischemia model, neonatal HI was induced in rat pups on postnatal day 7 (Figure 1). Rats were stimulated electrically at 2 Hz (1, 3, or 5 mA; 20 min), 3 times a week for 3 weeks (Figure 1). We evaluated spontaneous locomotor behavior and anxiety (Figure 2). There was a significant increase in the total distance traveled and velocity in the open field test in all EA-treated groups (EA1, EA3 and EA5) at 5 weeks after HI when compared with the results of the HI group but no change at 4 weeks (Distance: Figure 2A, F _(4,20)_ = 3.499, *p* = 0.025; velocity: Figure 2B, F_(4,20)_ = 5.073, *p =* 0.005). In addition, we analyzed the number of entries to the center zone to assess anxiety-like behavior. While the number of entries to the center zone reduced in the HI group, it significantly increased in the EA-treated group, indicating that EA treatment reduces anxiety-like behavior (Figure 2C).

The number of cylinder wall contacts with the impaired forelimb in the spontaneous cylinder test was significantly increased in all EA-treated groups at 4 and 5 weeks compared with the results in the HI group, with the greatest improvement in the EA3 group (Figure 2D, 4 weeks: F_(4,20)_ = 35.582, *p* < 0.001; 5 weeks: F_(4,20)_ = 50.421, *p* < 0.001). In the accelerated rotarod test, all EA-treated groups showed a significantly longer mean time on the rotarod at 4 and 5 weeks than did the HI group (Figure 2E, 5 weeks: F_(4,20)_ = 14.845, *p* < 0.001). In the passive avoidance test, which evaluated short-term learning and memory, the HI group showed significantly decreased retention latencies when compared with the sham group. Interestingly, retention latency was significantly increased in the EA3 group compared with that in the HI group (Figure 2F). Altogether, these results suggested that EA treatment reversed motor and memory dysfunction following neonatal HI. The effects were the strongest on anxiety-like behavior, spontaneous limb use, and short-term memory, compared with other treatments using electric stimulation with 3-mA intensity (EA3 group).

### 3.2. Effects of EA Treatment on the Corpus Callosum Thickness, Oligodendrocytes, and Astrocytes

**Figure 3:**
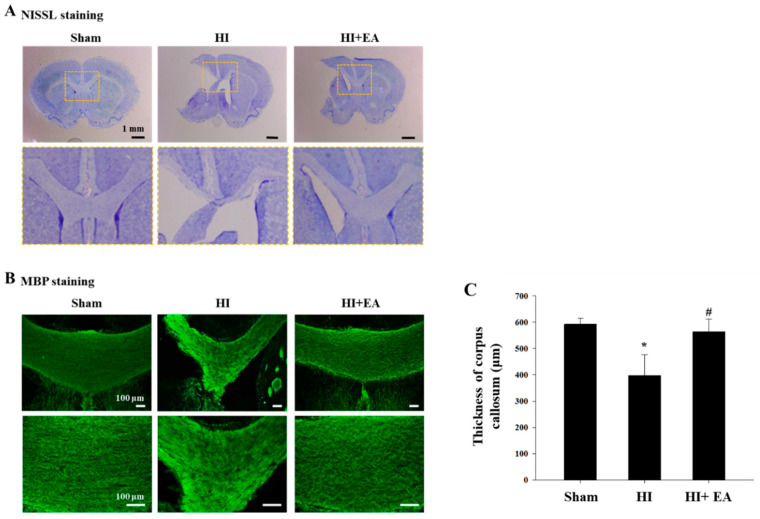
Effect of EA treatment on the corpus callosum following HI. (**A**) NISSL staining to show overview images of the sections. Corpus callosum in dotted-square area was observed. Magnification: ×8, scale bar = 1 mm. (**B**,**C**) Immunofluorescence staining for myelin basic protein (MBP). EA (3-mA) treatment resulted in a marked increase in the thickness of the corpus callosum compared with the results in the HI group. Magnification: ×100 (top), ×200 (bottom); scale bar = 100 μm. (**C**) The thickness of the corpus callosum was quantified using the i-solution software. Data are presented as mean ± SEM. * *p* < 0.05 vs. sham, ^#^ *p* < 0.05 vs. HI. sham (n = 6), HI (n = 6), HI+EA (n = 6).

The efficacy of the EA3 group (EA 3 mA) was the highest in all EA groups; therefore, we used the EA 3-mA treatment to compare the EA groups with the HI and sham groups in all subsequent experiments. We measured the thickness of the corpus callosum after staining for myelin basic protein (MBP) (Figure 3) and found it to be significantly decreased in the HI group when compared with that in the sham group; significant recovery in thickness was observed in the EA group (Figure 3C, F_(2,10)_ = 5.435, *p* = 0.025).

**Figure 4:**
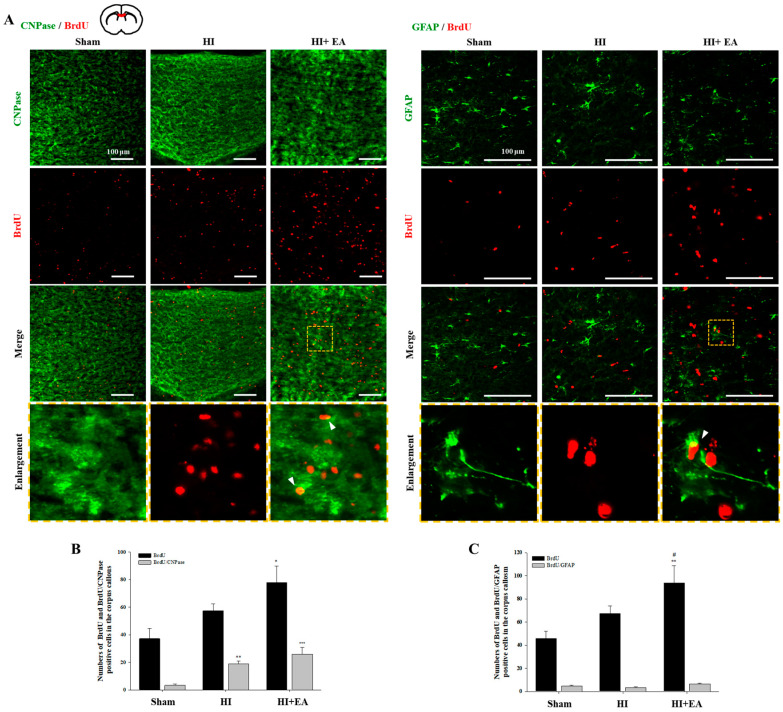
Effect of EA on the oligodendrocytes and astrocytes in the corpus callosum. (**A**) Immunofluorescence staining against CNPase (green) and BrdU (red) after EA (3 mA) treatment (top). Magnification: ×200; scale bar = 100 μm. Immunofluorescence of GFAP (green) and BrdU (red) after EA (3 mA) treatment (bottom). Magnification: ×400; scale bar = 100 μm. (**B**,**C**) Immunofluorescence-positive cells were quantified in the corpus callosum. The number of BrdU^+^ cells in the HI + EA group was significantly increased as compared with that in the HI group. The number of CNPase^+^ /BrdU^+^ cells in the HI + EA group was increased as compared with that in the HI group, although this difference was not significant. Data are presented as mean ± SEM (n = 6 per group). * *p* < 0.05, ** *p* < 0.01 and *** *p* < 0.001 vs. sham, ^#^ *p* < 0.05 vs. HI.

We then observed the BrdU^+^ cells and measured the colocalization of BrdU with 2,3-cyclic nucleotide-3-phosphodiesterase (CNPase), a marker of immature and mature myelinating oligodendrocytes [23] or glial fibrillary acidic protein (GFAP), an astrocyte marker, in the corpus callosum (Figure 4A). There was a significant increase in BrdU^+^ cells in the corpus callosum in the EA-treated group (Figure 4B,C, BrdU^+^: F_(2,8)_ = 7.741, *p* = 0.013; BrdU^+^: F_(2,8)_ = 12.190, *p* = 0.004). EA treatment increased the number of CNPase^+^/BrdU^+^ cells (Figure 4B, F_(2,10)_ = 17.212, *p* < 0.001). A similar result was observed in colocalization of BrdU with oligodendrocyte transcription factor 2 (Olig2) or Olig2 with anti-adenomatous polyposis coli clone CC1 (CC1) in the corpus callosum (Supplementary Figure S1, Olig2^+^/CC1^+^: F_(2,15)_ = 13.489, *p* < 0.001). In contrast, there were very few GFAP^+^/BrdU^+^ cells with no significant difference between groups (Figure 4C, F_(2,10)_ = 4.040, *p* = 0.052).

### 3.3. Effects of EA Treatment on Neurogenesis in the Subventricular Zone and Hippocampus

**Figure 5:**
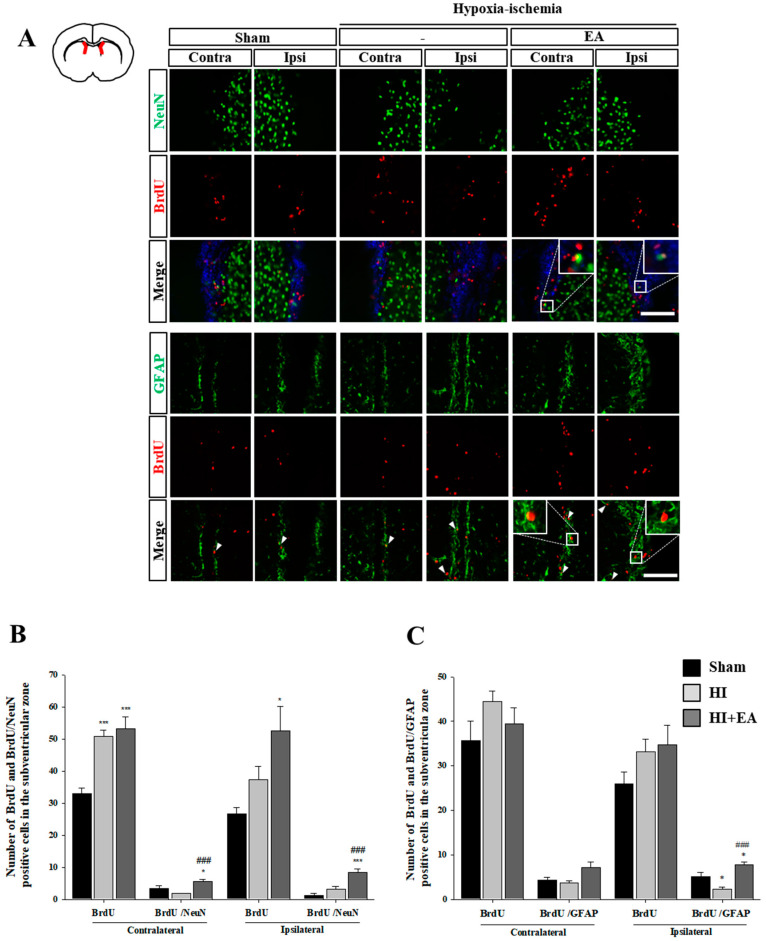
Effect of EA on the activation of neurons and glial cells in the subventricular zone. ((**A**), top) Immunofluorescence of NeuN (green) and BrdU (red) after EA (3 mA) treatment. Nuclear DNA was counterstained with DAPI (blue). Scale bar = 100 µm. ((**A**), bottom) Immunofluorescence of GFAP (green) and BrdU (red) after EA (3 mA) treatment. Scale bar = 100 µm. (**B**) The number of NeuN^+^/ BrdU^+^ cells in both hemispheres was significantly higher in the EA group than in the HI group. Data presented as mean ± SEM. * *p* < 0.05 and *** *p* < 0.001 vs. sham, ^###^ *p* < 0.001 vs. HI. (**C**) The number of GFAP^+^/BrdU^+^ cells in ipsilateral hemisphere was significantly higher in the EA group than in the HI group. Data are presented as mean ± SEM. * *p* < 0.05 vs. sham, ^###^ *p* < 0.001 vs. HI.

**Figure 6:**
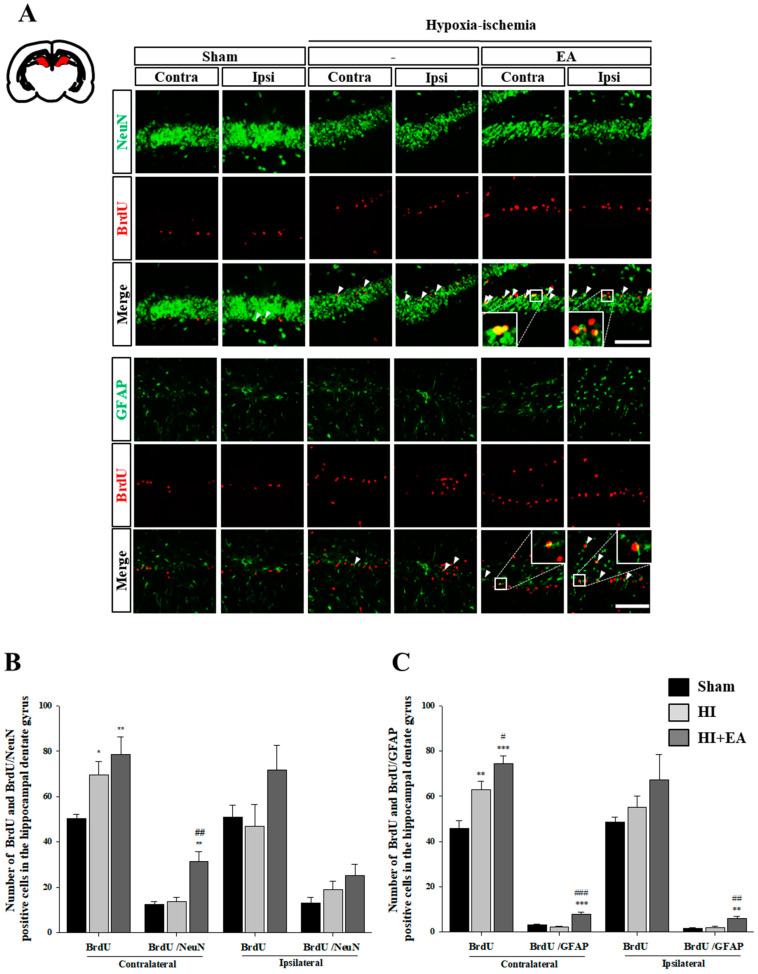
Effect of EA on the activation of neurons and glial cells in the dentate gyrus. ((**A**), top) Immunofluorescence image of NeuN (green) and BrdU (red) after EA (3 mA) treatment. NeuN^+^/BrdU^+^ cells (arrowheads). Scale bar = 100 µm. (A, bottom) Immunofluorescence of GFAP (green) and BrdU (red) after EA (3 mA) treatment. GFAP^+^/BrdU^+^ cells (arrowheads). Scale bar = 100 µm. (**B**) The number of NeuN^+^/BrdU^+^ cells in the contralateral hemisphere was higher in the EA group than in the HI group. Data presented as mean ± SEM. * *p* < 0.05, vs. sham, ^##^ *p* < 0.01 vs. HI. (**C**) The number of GFAP^+^/BrdU^+^ cells in both hemispheres was significantly higher in the EA group than in the HI group. Data are presented as the mean ± SEM. ** *p* < 0.01, and *** *p* < 0.001 vs. sham, ^#^ *p* < 0.05, ^##^ *p* < 0.01 and ^###^ *p* < 0.001 vs. HI.

We measured the number of BrdU^+^ cells in the subventricular zone and dentate gyrus of the hippocampus and found that the EA group had an increase in the total number of BrdU^+^ cells in both regions. Especially, there was a significant increase in BrdU^+^/neuronal nuclei (NeuN)^+^ cells in the EA group compared with that in the contralateral (Figure 5A,B, F_(2,10)_ = 9.925, *p* < 0.004) and ipsilateral (Figure 5A,C: F_(2,10)_ = 31.582, *p* < 0.001) HI groups. In addition, there was a significant increase in BrdU^+^/GFAP^+^ cells in the ipsilateral subventricular zone in the EA group compared with that in the HI group (Figure 5A,C, F_(2,10)_ = 18.484, *p* < 0.001). We also found a significant increase in the number of BrdU^+^/NeuN^+^ cells and BrdU^+^/GFAP^+^ cells in the EA group compared with that in the sham or HI group in the dentate gyrus (BrdU^+^/NeuN^+^: Figure 6A,B, F_(2,15)_ = 14.355, *p* < 0.001; BrdU^+^/GFAP^+^: Figure 6A,C, contralateral: F_(2,10)_ = 32.872, *p* < 0.001, ipsilateral: F_(2,6)_ = 16.158, *p =* 0.004). These results suggested that EA might promote the proliferation and differentiation of neural stem cells in the subventricular zone and dentate gyrus following HI.

## 4. Discussion

Infants who have been exposed to HI insults exhibit neurodevelopmental impairments with life-long chronic disabilities that affect sensorimotor deficits as well as impairments in learning and memory [24,25,26]. These pathological processes include damage to the neuronal cell population in the basal ganglia and cortex as well as significant cellular necrosis and axonal damage in the cerebral white matter, which manifests principally in motor and memory deficits [27]. First, we performed behavioral tests to assess motor and memory function following EA treatment and found that EA ameliorated motor dysfunction and memory impairments following neonatal HI (Figure 2), suggesting that EA may be beneficial as a therapy for cerebral palsy. Moreover, EA treatment with 3 mA intensity significantly enhanced functional outcomes, including a reduction in anxiety-like responses, and led to recovery of limb use and memory dysfunction, compared with EA at other intensities (Figure 2); therefore, we used this intensity for further experiments.

White matter injury in prematurely-born children leads to neuronal dysfunction, sensorimotor dysfunction, and cognition impairments, which are found in many forms of cerebral palsy [28,29]. Myelin is an essential insulator of neuronal axons, which is crucial for proper neuronal activity. Oligodendrocytes, the myelinated cells in the brain, are the cellular targets of injury in neonates and their damage may lead to cerebral palsy [30,31]. White matter injury in the corpus callosum leads to neuronal dysfunction and behavioral deficits in cerebral palsy [28,30]. We assessed damage to the corpus callosum by measuring its thickness because atrophy of these connecting tracts is correlated with neuronal deficits [32,33]. We measured the thickness of the corpus callosum between groups (Figure 3) and found that treatment with EA at 3 mA intensity increased the thickness of the corpus callosum, suggesting that this therapy may restore myelin.

The generation of oligodendrocytes, promotion of oligodendrogenesis, and promotion of myelin sheath thickness with myelin remodeling is associated with improved motor function in the subcortical white matter [34,35]. Therefore, we investigated whether alterations in newly generated oligodendrocytes were associated with alterations in corpus callosum thickness (Figure 4). We observed significant increases in the number of BrdU^+^ proliferating cells and small increases in the number of new oligodendrocytes following EA treatment. Oligodendrocyte progenitor cells in the damaged brain proliferate and migrate radially to the neighboring white matter tracts such as the corpus callosum and differentiate into mature myelinating oligodendrocytes, subsequently leading to restoration of myelin sheaths [9,36]. Therefore, our results suggested that EA-induced new oligodendrocytes from oligodendrocyte progenitor cells may drive the amelioration of HI-induced thickness loss of the corpus callosum.

In the adult mammalian brain, neuronal replacement by neural stem cells occurs via neurogenesis after focal and global cerebral ischemia in both the subventricular zone and dentate gyrus [10,12]. Thus, neurogenesis is important for the compensation and recovery of lost neurons and functions. Enhancing neurogenesis in two restricted sites, the subventricular and subgranular zones of the dentate gyrus may ameliorate functional impairment [11,37]. Astrocytes may play critical roles in regulating neurogenesis in both intact and post-injury adult brains [38]. Under certain circumstances, neurogenesis and gliogenesis interactions facilitate the transformation of glial cells into neurons. Therefore, modulating the balance between neurogenesis and gliogenesis may aid in neuro-restoration in diseases [39]. Our study showed that EA treatment significantly increased the number of newly generated neurons and astrocytes in these two regions (Figure 5 and Figure 6). These results suggested that EA treatment promotes neurogenesis and gliogenesis in both regions and may accelerate functional recovery after HI.

Some studies have reported that electric stimulation on the scalp, such as acupuncture and transcranial current stimulation, accelerates the recovery of function and neurogenesis. Various acupoint-based treatments are used in scalp electric acupuncture (for example, Baihui [GV20]). This improves neurological function scores via cell proliferation and differentiation [14,17,40,41,42]. Transcranial direct current stimulation (tDCS) induces neurogenesis, independently of polarity and cathodal tDCS-recruited oligodendrocyte precursors, toward the lesion in a rat focal cerebral ischemic model [43]. Electric stimulation increases neural stem cell migration toward the lesion through the NMDAR/Rac1/actin axis [44] and increases the migration of ventral midbrain-derived dopaminergic neural progenitor cells via Wnt/GSK3β signaling [45]. Moreover, electric stimulation facilitates the differentiation of neural stem cells into mature neurons via interferon-γ, a neuronal maturation factor [46]. Previous acupoint-based treatments following neonatal HI involved the stimulation of a specific point of the scalp (e.g., Baihui, the midpoint of the line connecting the apexes of both the ears on the parietal bone) [14,18], but EA stimulates a wider area on the scalp over the motor cortex. The difference between these two methods makes it difficult to compare the current results with those from our previous experiments; however, EA leads to somewhat better behavioral recovery, forelimb movement, and motor coordination and balance than acupoint-based treatments, suggesting that it is a better intervention for endogenous neurogenesis [14,18]. Furthermore, studies have shown that transcranial current stimulation, which applies a direct current in both anodal and cathodal directions, induces neurogenesis from the subventricular zone and recruits oligodendrocyte precursors toward lesions [43,47]. There may be differences in the neurobiological mechanisms of electric stimulation to the scalp: acupuncture is localized via needle stimulation, whereas transcranial currents induce direct excitability of the brain cortex [40,48]. However, common molecular mechanisms may be involved as both therapies induce beneficial behavioral effects via multifaceted mechanisms [43,47,49]. Studies have shown that electrical stimulation can induce the release of neurotrophic factors in the brain, for example brain-derived neurotrophic factor (BDNF). Therefore, BDNF and tropomyosin-related kinase B signaling pathways may have beneficial roles in functional recovery via the stimulation of neurogenesis following electric acupuncture and transcranial current stimulation [21,49,50,51]. A greater understanding of the neural responses to electric stimulation on the scalp is critical before this therapy can be offered to patients with conditions affecting the central nervous system [21]. Our study shows that EA therapy was effective at ameliorating HI-induced behavioral deficits via the activation neural stem cells in a cerebral palsy-like rat HI model, and this effect was more pronounced with 3 mA stimulations (Figure 2).

## 5. Conclusions

In conclusion, currently, there are no effective or novel treatments for cerebral palsy; therefore, we propose that engrafted alternative current stimulation on the scalp projection location of the motor cortex by filiform needle offers a therapeutically relevant option to support the functional improvement of motor deficits and memory impairments in perinatal HI causing neurologic complications such as cerebral palsy. Our study provides new insight into the therapeutic treatment of brain injury in newborns following HI and suggests further combination treatments.

## Supplementary Materials

The following are available online at https://www.mdpi.com/2075-1729/10/10/240/s1, Figure S1: Effect of EA on the activation of oligodendrocytes in the corpus callosum.
