# Supplementary material for: Electroacupuncture on the Scalp over the Motor Cortex Ameliorates Behavioral Deficits Following Neonatal Hypoxia-Ischemia in Rats via the Activation of Neural Stem Cells
Source: Life (Basel). 2020 Oct 14;10(10):240. doi: 10.3390/life10100240 (PMC7602251; doi:10.3390/life10100240)
Supplement: Supplementary file 1 [file life-10-00240-s001.pdf]

life

MDPI

Supplementary Materials

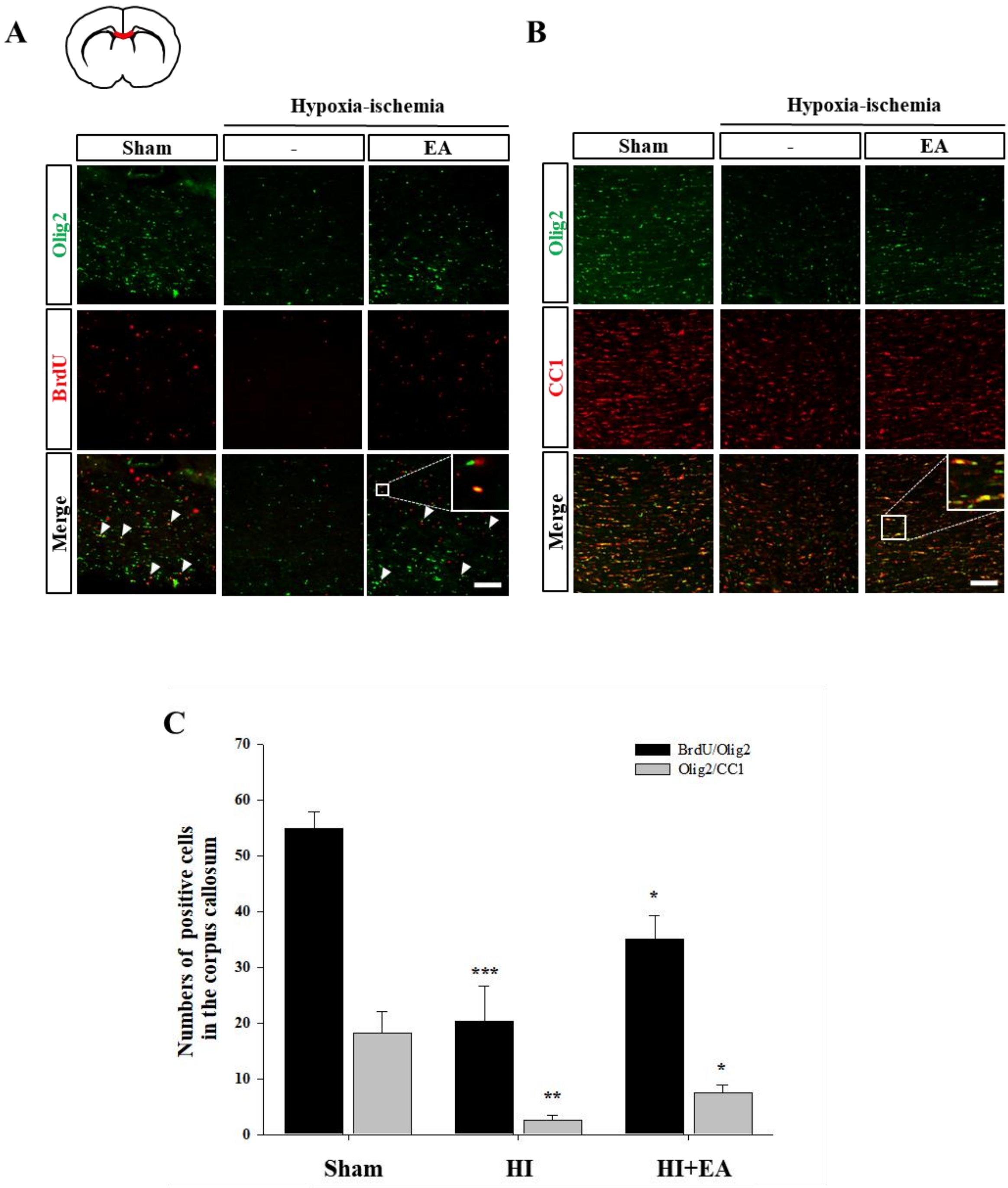

Figure S1. Effect of EA on the activation of oligodendrocytes in the corpus callosum. **(A)** Fluorescence staining against Olig2 (green) and BrdU (red) after EA (3mA) treatment. Scale bar = 100 μm. **(B)** Immunofluorescence staining against Olig2 (green) and CC1 (red) after EA (3mA) treatment. Scale bar = 100 μm. **(C)** The number of Olig2^{+}/BrdU^{+} cells and Olig2^{+}/CC1^{+} cells. Olig2^{+}/BrdU^{+} cells and Olig2^{+}/CC1^{+} cells were increased in the EA group. Data presented as mean ± SEM. $^{*}P < 0.05$, $^{**}P < 0.01$ and $^{***}P < 0.001$ *vs*. sham. Olig2 (NBP1-28667, Novus Biologicals, Littleton, USA); and CC1 (GTX16794, APC antibody, Gentex Corp., CA, USA).
